# Early potential safety signals for gliptins and gliflozins using real-world pharmacy data compared to spontaneous reporting

**DOI:** 10.1371/journal.pone.0352399

**Published:** 2026-06-25

**Authors:** Maria-Isabel Jimenez-Serrania, Sara-Maria Martinez-Gonzalez, Javier Herradon-Muñoz, Carlos Treceño-Lobato

**Affiliations:** 1 ADViSE Research Group, Department of Health Sciences, Faculty of Health Sciences, Miguel de Cervantes European University (UEMC), Valladolid, Spain; 2 Council of Professional Colleges of Pharmacists of Castilla y León, Castilla y León, Valladolid, Spain; University of Diyala College of Medicine, IRAQ

## Abstract

**Background:**

Dipeptidyl peptidase 4 inhibitors (DPP4i, gliptins) and sodium-glucose co-transporter 2 inhibitors (SGLT2i, gliflozins) are two oral antidiabetic drug classes that have been widely used in recent decades. Their pharmacological actions have led to the identification of new adverse drug reactions and contraindications. In this context, ambulatory real-world data (RWD) analysis has the potential to detect early signals of suspected adverse drug reactions (ADR) that complement or anticipate risk events.

**Methods:**

We conducted an exploratory pharmacovigilance study based on a multicentre observational cross-sectional survey with retrospective 6-month recall in community pharmacies among patients treated with DPP4i or SGLT2i, using metformin as a control. Candidate drug–event signals identified in the pharmacy dataset collected in 2021 were compared with spontaneous reporting data from the Spanish Agency of Medicines and Medical Devices repository in 2022 and 2024. A validated adaptation of the Bayesian Confidence Propagation Neural Network (BCPNN) methodology was applied in all analyses.

**Results:**

Exploratory signals identified in the community pharmacy dataset included sitagliptin–dry mouth (FDR 0.059), empagliflozin–asthenia (0.067), linagliptin–bone fracture (0.077), linagliptin–renal impairment (0.082), dapagliflozin–hyperglycemia (0.088), canagliflozin–pruritus (0.092), dapagliflozin–urinary tract infection (0.096), and alogliptin–exanthematic eruptions (0.100). Some of these findings were already described in the Summaries of Product Characteristics (SmPC), whereas others were not and later showed greater convergence with spontaneous reporting patterns in 2024. These findings should be interpreted as exploratory statistical signals for follow-up, particularly when based on small numbers of reports.

**Conclusions:**

Community pharmacy real-world data may contribute to the early identification of potential safety signals that complement spontaneous reporting systems. Signals detected near the exploratory threshold should be interpreted cautiously and considered candidates for monitoring and further investigation rather than confirmed adverse drug reactions.

## Introduction

The management of type 2 diabetes (T2D) has evolved significantly with two relatively new oral hypoglycemic drugs that target different pathways in glucose metabolism: Dipeptidyl Peptidase-4 inhibitors (DPP4i) and Sodium-glucose cotransporter 2 inhibitors (SGLT2i) have gained prominence due to their unique mechanisms of action and beneficial effects on glycemic control and other health outcomes. SGLT2 inhibitors have become a key component in the management of diabetes and cardiovascular disease.

DPP-4i contribute to better glycemic control, enhancing insulin secretion and suppressing glucagon release. This glucose-lowering group of drugs inhibits DPP-4 enzyme and increase concentrations of incretins glucagon like peptide-1 (GLP-1) and glucose-dependent insulinotropic polypeptide (GIP). They are generally well-tolerated, without significant weight gain or high risk of hypoglycemia [1]. The most frequent adverse events reported in DPP4i include mild to moderate symptoms such as constipation, nasopharyngitis, urinary tract infection, myalgia, arthralgia, headache and dizziness [1,2].

SGLT2i increase urinary glucose excretion by inhibiting glucose reabsorption in the renal proximal tubule, lowering blood glucose levels. [3]

SGLT2i show an appropriate tolerability profile with minimal adverse effects, mainly associated with their mechanism of action. These include genitourinary tract infections, dehydration and other associated symptoms irrespective of a chronic condition and sort of SGLT2 inhibitors used. [4,5]

An SGLT2i has a low risk of hypoglycemia, only increased when it is added to agents that cause hypoglycaemia, such as insulin or sulphonylureas. [6]

Drug-induced adverse events can lead to hospitalization, cause physical or mental damage even death to patients. It is of utmost importance to identify and detect adverse-drug reactions (ADRs) over time to assure a safe use of pharmacological treatment. [7]

The principal methods for pharmacovigilance consist of post-marketing surveillance studies and the mining of spontaneous adverse event reporting systems. The spontaneous notification systems in Pharmacovigilance have large databases and are mainly focused on the early detection of adverse reactions of commercialized drugs with cumulative data over the time. [8]

Also, spontaneous reporting is more efficacious when adverse events are rare and uncommon (less than 1% of treated patients) and when the event is typical of a drug-induced condition. Their use is limited to detecting a small increase in the rate of common events. [9]

A safety signal refers to information on a new or known side effect that may be caused by a medicine and is based on accumulated information from different sources. It’s important to note that the presence of a safety signal does not indicate a direct causal relationship between a side effect and a medicine. Rather, it offers a hypothesis that, supported by data and arguments, justifies the need for further assessment. [10]

In the past, this signal detection was based on a case-by-case analysis. In recent years, data mining techniques have become a more efficient method, understanding it as an analysis of data from different sources such as Pharmacovigilance system databases and real-world healthcare databases. The comparison of these two types of data is showing an interesting potential in prospective pharmacovigilance signal detection [11].

Modern signal generation has the capability to identify early potential indicators of harm and alert clinicians to potential novel therapeutic risks. These automated methods use algorithms to discover unexpected events within large and entire Pharmacovigilance databases. These algorithms analyze how much the number of observed cases through notifications deviates from the expected cases. In other words, they calculate estimators of the disproportionality of reports. [12,13]

That is why, currently, in addition to the alerts generated in the Regional Pharmacovigilance Centres, active searches for signals can be carried out using these automated methods.

### Objectives

The aim of this study was twofold: (1) to identify early safety signals in patients using Dipeptidyl peptidase 4 inhibitors (DPP4i, gliptins) and Sodium-glucose co-transporter 2 inhibitors (SGLT2i, gliflozins) in the real-world setting; and (2) to evaluate whether structured community pharmacy data can anticipate signals later observed in spontaneous reporting systems.

## Materials and methods

### Design

We first conducted an exploratory pharmacovigilance analysis using drug–event pairs derived from a multicentre observational cross-sectional survey conducted in 2021 in community pharmacies. The survey included retrospective patient recall of adverse reactions experienced during the previous 6 months among users of DPP4i (ATC A10BH) and SGLT2i (ATC A10BK) marketed in Spain. Biguanides (ATC A10BA), represented by metformin (ATC A10BA02), were used as a control group because metformin is a widely used first-line treatment for type 2 diabetes with a well-established safety profile.

Secondly, we replicated a homologous analysis using spontaneous reporting data from national Spanish Agency of Medicines and Medical Devices accumulative repository to contrast and validate our results in RWD for 2022 (immediately after 2021 study, hoping to observe similarities in events reported in the last year) and 2024. We likewise analysed reported data of all drug-event pairs related to the chemical subgroups A10BH, A10BK, and A10BA02 (metformin) as control. Data below the heading Preferred Term (PT) of adverse drug reactions for each active ingredient of interest were extracted on January 12, 2022 and September 2, 2024.

### Setting

For real-life ambulatory analysis, we used information obtained through an observational, multi-centre, cross-sectional survey that was carried out at the 170 pharmacies that integrate the Sentinel Surveillance Network of Pharmacies in Castilla y León, Spain.

No formal sample size calculation was performed, as the study was conceived as an exploratory signal detection analysis within the operational capacity of the sentinel pharmacy network.

Each pharmacy in the network selected 9 patients receiving chronic treatment for more than 6 months: 3 with DPP4i, 3 with SGLT2i, and 3 with metformin as the only antidiabetic drug. Inclusion criteria were adults (≥18 years) with type 2 diabetes receiving metformin alone, a DPP4 inhibitor alone, or an SGLT2 inhibitor alone for at least 6 months. Patients receiving additional glucose-lowering therapies (e.g., insulin, sulfonylureas, GLP-1 receptor agonists, or other oral antidiabetic agents) were excluded to facilitate interpretable drug–event signal detection. Non-antidiabetic concomitant medications could be recorded during the interview but were not incorporated into the disproportionality analysis due to the difficulty to attribute and compare with spontaneous reporting.

Participants were capable of understanding and completing the questionnaire and of providing written informed consent. Participant recruitment was conducted between January 1, 2021, and December 31, 2021.

**Fig 1:**
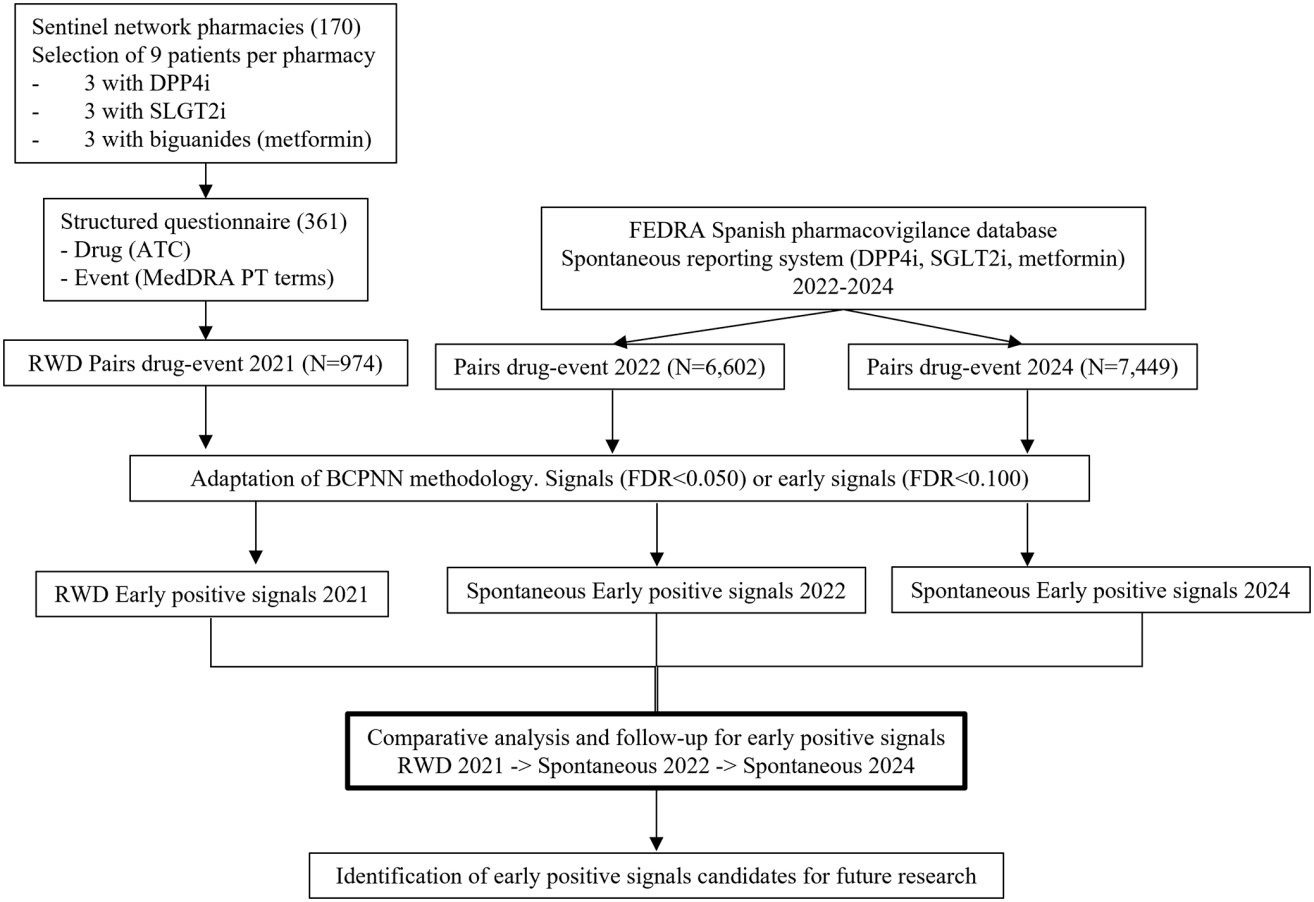
Flowchart of the study procedure: RWD analysis, Spontaneous reporting follow-up analysis and comparison.

The final sample comprised patients who accept to participate, and information was gathered using a structured questionnaire that included any adverse reactions the patient had experienced in the preceding 6 months (S1 File) Furthermore, any associated adverse reactions, signals or symptoms that the pharmacist was able to collect during the interview, even if they were not included on the questionnaire, were recorded as observational. All the pharmacists participating in the study had to pass a prior accreditation course on pharmacovigilance procedures and were trained in the notification of suspicions following the same procedure of reporting to the Spanish Agency for Medicines and Medical Devices (AEMPS). In fact, all suspected adverse reactions detected must be reported to the national system. Each case is strictly codified by pharmacists to avoid duplications. For the present signal detection analysis, the working dataset provided only contains drug–event pairs (S1 Dataset, tab RWD_extract); individual-level covariates were not available for adjustment in the disproportionality analysis. Therefore, the study could not control for age, sex, renal function, diabetes duration, comorbidities, dose, treatment duration, or concomitant medications. Raw survey data were pre-processed case by case into drug (ATC code)–event pairs by the statistical experts of the research group (see flowchart, Fig 1). Only pairs with ≥1 occurrence were retained. We obtained a list of active ingredient-suspected event pairs and number of times repeated.

For the comparative analysis, we used data from the FEDRA (Farmacovigilancia Española Datos de Reacciones Adversas) database of the Spanish Agency for Medicines and Medical Devices (AEMPS), which contains spontaneous reports of suspected adverse drug reactions submitted by healthcare professionals, patients, and pharmaceutical companies.[14]

The reports are reviewed and validated by pharmacovigilance professionals within the regional centers of the Spanish Pharmacovigilance System (SPS) [14]. SPS has operated since nearly 30 years and allows all healthcare professionals to notify any suspected ADRs, particularly in the cases in which the adverse reaction is serious or the suspected drug is a medication subject to special surveillance or recently commercialised in Spain. All suspected ADRs were categorized using MedDRA terminology [15]. The database includes information such as patient demographics, suspected drugs, concomitant medications, adverse events, seriousness reported, and outcome aggregated by System Organ Class (SOC). But only information of number of cases of suspected adverse reactions reported by active ingredient is disaggregated by Preferred Term (PT). No causality assessment is included, only reports. Prior to accessing these data, it must be confirmed that the information contained in the database cannot be used by healthcare professionals to make decisions about a patient’s choice of treatment. The information collected in this database cannot be used to calculate the frequency of adverse reactions in patients taking the medication, nor can it establish causality or make comparisons regarding the safety of different medications. Although it does not confirm causality, it is a valuable tool for detecting safety signals and monitoring drug safety in real-world conditions. It is important to note that the validated cases reported to FEDRA are also submitted to EudraVigilance database. These data are considered reliable for pharmacovigilance research and signal generation.

Data for every active ingredient were processed and merged in a common database of pairs drug-event for 2022 and 2024 (see flowchart, Fig 1). Only pairs with ≥1 occurrence were retained. We obtained a list of active ingredient-suspected event pairs and number of times repeated.

### Statement on Availability of data and materials

The spontaneous reporting data used in this study are publicly available from the Spanish Agency of Medicines and Medical Devices (AEMPS) repository.

The anonymized working dataset derived from the community pharmacy study is provided as Supporting Information file (S1 Dataset), containing aggregated drug–event pair data to reproduce the signal detection analyses (tab extract_RWD), and results obtained in BCPNN analysis (tab RWD_analysis).

The original individual-level dataset collected through the sentinel pharmacy network was obtained under institutional authorization from the Council of Professional Colleges of Pharmacists of Castilla y León and cannot be publicly shared due to legal, ethical, and governance restrictions. However, the shared anonymized supplementary dataset contains the information necessary to reproduce the analyses presented in this study.

### Statistical Analysis with Bayesian methodology

In all analyses (RWD 2021, FEDRA 2022, FEDRA 2024) we performed an adaptation of the Bayesian methodology applied by the Uppsala Monitoring Centre belonging to WHO for searching early signals in databases of suspected adverse drug reaction reports.

The BCPNN method was originally developed and applied to the WHO international pharmacovigilance database [16], demonstrating its robustness in managing large-scale spontaneous report data. Unlike traditional disproportionality measures, BCPNN incorporates Bayesian inference, allowing for more stable signal detection even in the presence of sparse or incomplete data. BCPNN offer greater signal specificity and reduced the likelihood of false positives compared to methods like the Proportional Reporting Ratio (PRR) and the Reporting Odds Ratio (ROR) as estimators of disproportionality [17]. Across simulated datasets, BCPNN showed a substantially lower proportion of false positives among the top-ranked 500 drug–event pairs. In some scenarios, it achieved a false positive rate as low as ~1%, compared to over 50% for the least effective methods. The Bayesian framework underlying BCPNN offers better performance with sparse data and yields more stable signal scores, especially in the presence of low event counts. Additionally, the capacity of the BCPNN method for the early detection of new adverse drug reactions (ADRs) has been widely demonstrated [12,13,18–21].

Nowadays, the original Bayesian Confidence Propagation Neural Network (BCPNN) was extended to the multiple comparison setting and is considered a validated tool to detect new ADR signals [8]. This method relies on the posterior likelihood of the null hypothesis (post.H0) and allows to indirectly obtain the false positive Bayesian estimator (FDR), which represents a measure for the detected signals. The original algorithm of BCPNN calculates how much differing the observed ADR reported (‘posterior probability’; post. H0) with respect to expected ADR (‘anterior probability’; arbitrary designed). The disproportionality estimator called information component ‘IC’ is obtained for each possible drug-ADR combination reported, and the threshold of a positive signal detected is a Q0.025IC>0). Moreover, the extension to a multiple comparison setting considers –instead of the classic BCPNN arbitrary limits- the own data reported to establish the positive signal detection threshold. This approach orders each drug-event based on the posterior probability of the null hypothesis of interest (post.H0), and indirectly obtains the Bayesian estimator of false discovery rate (FDR), that works like a p-value, offering a positive signal if FDR < 0.05 [8].

Later adaptations of this methodology can be valuable and trustworthy with a correct interpretation of the signals [22]. The adaptation applied in this study consists in contrasting all the events of specific Anatomical Therapeutic Chemical (ATC) Classification System subgroups [23] that share their recent commercialization as common factor, isolated from the integral database with all active ingredients of all subgroups, and including a control. In this case, the chemical subgroups A10BH and A10BK were considered in the analysis and A10BA02 metformin was considered as control. This adaptation had been previously applied and shown to be robust and consistent with other specific groups of drugs. [24–26]

Statistical analyses were conducted using R version 3.4.1 (R Foundation for Statistical Computing, Vienna, Austria) and the PhViD package version 1.0.8 for pharmacovigilance signal detection.

In our analysis, we performed the algorithm with the following arguments: value of the relative risk (RR) proven to be higher than 1 (RR < 1); minimum number of cases per pair [drug-event] in order to be potentially considered as a signal (N = 1); rule of decision for the generation of signals: FDR; limit or threshold for the decision rule: FDR > 0.05; statistics used for ordering the drug-event pairs: posterior probability of the null hypothesis (post.H0); calculation of the distribution of the statistic of interest by: approximation to the normal distribution and signal strength assessed using empirical estimation through Monte Carlo simulations (NB.MC = 10,000 iterations). Details of the package and algorithm performed are reported in S2 File.

To extent the analysis, we extracted all signals and proposed as early positive signals those with FDR into the range 0.05-0.10, due to FDR works like a p-value. An FDR or p-value ≤ 0.10 is not a universally accepted standard of statistical significance, but it may be relevant in certain contexts, such as exploratory research or when seeking to avoid type II errors, failing to reject the null hypothesis when it is false.

We applied a p-value threshold of < 0.10 as an exploratory criterion for early signal detection using the extended BCPNN model. In fact, the introduction of Bayesian signal detection approaches within a multiple-comparison framework, noting that FDR control is flexible and depends on the desired trade-off between sensitivity and specificity [12]. From this perspective, using a p-value < 0.10 increases sensitivity at the exploratory stage, without compromising the overall interpretation or requiring formal hypothesis testing.

Due to this extension, if a signal presents an FDR into the range 0.05-0.10, we will consider it as an early positive signal to follow up, provided it also fulfils high specificity (Sp≥0.99) and false negative rate FNR < 49%, which implies that, at least, half of the rejected signals are truly negative.

In results, we present positive signals obtained from each drug-event pair for the RWD analysis ordered by FDR, and including values of number of pairs (N), False negative rate (FNR), sensitivity (Se), and specificity (Sp) (Table 1). According to whether signal is reported in fact sheets or not, we propose a analysis in seven different groups (see definition details for each group in Results).

**Table 1:** Early safety signals for gliptins and gliflozins obtained in real-life ambulatory (RWD) setting and classification depending on fact sheet reporting.

ATC code	Active ingredient	PT MedDRA	N	FDR	FNR	Se	Sp	Group^a^
A10BA02	metformin	diarrhea	30	0.053	0.474	0.008	1.000	(control)
A10BH01	sitagliptin	dry mouth	12	0.059	0.472	0.017	1.000	3
A10BK03	empagliflozin	asthenia	8	0.067	0.470	0.025	0.999	6
A10BH05	linagliptin	bone fracture	4	0.077	0.468	0.033	0.998	7
A10BH05	linagliptin	renal impairment	4	0.082	0.467	0.040	0.998	7
A10BK01	dapagliflozin	hyperglycemia	7	0.088	0.465	0.048	0.997	6
A10BK02	canagliflozin	urticaria pruritus	4	0.092	0.463	0.056	0.996	4 5
A10BK01	dapagliflozin	urinary tract infections	7	0.096	0.461	0.064	0.995	1
A10BH04	alogliptin	exanthematic eruptions	2	0.100	0.459	0.071	0.994	2

PT, Preferred Term according to the MedDRA classification; FDR, Bayesian False Discovery Rate estimator; FNR, Bayesian False Negative Rate estimator; Se, selectivity; Sp, specificity. ^**a**^**Group:** (1) Safety traces stated as ADR frequent in fact sheets and, also, stated for other active ingredients of their group. (2) Safety traces stated as ADR frequent in fact sheets; but unknown frequency with other active ingredients of their group. (3) Safety traces stated as ADR are uncommon in fact sheets and not reported with other active ingredients of the group. (4) Safety traces stated as infrequent in fact sheets and not reported with other active ingredients of the group. (5) Safety traces not reported as ADR in fact sheets but stated for other active ingredients of the group. (6) Safety traces not reported as ADR in fact sheets. (7) Safety traces not reported as ADR in fact sheets.

**Fig 2:**
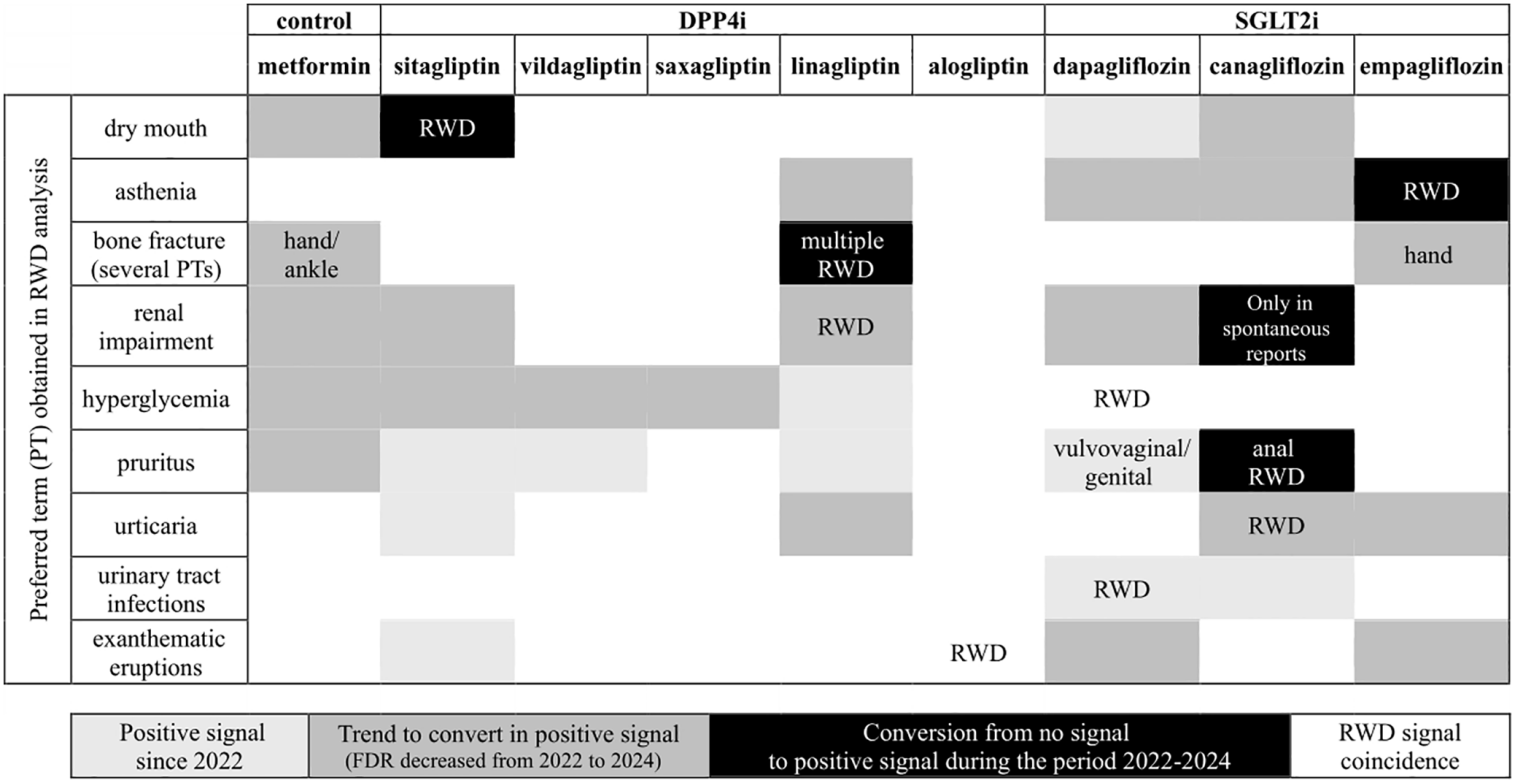
Heat map of safety signals obtained in real-life ambulatory (RWD) study followed up in spontaneous reporting database throughout the period 2022-2024.

Due to the extension of results in spontaneous reporting, we present data and a comparative follow-up as a supplementary material (S1 Table) and summarized this information considering the previous RWD positive signals in a heatmap (Fig 2).

Aggregation of patient characteristics by System Organ Class (SOC) in the spontaneous reporting database do not allow for including resume and comparative analysis between both databases.

### Statement of Informed Consent and Institutional Review Board Approval

This study involved human participants and was approved by the Research Ethics Committee for Medicines (CEIm) of the Valladolid East Health Area (approval reference CON-DAP-2019-01; local code EPA 19–322) on 23 January 2020. The study was conducted in accordance with the Declaration of Helsinki and applicable national regulations. Written informed consent was obtained from all participants before inclusion. For the analysis of spontaneous adverse drug reaction reports obtained from the Spanish Agency of Medicines and Medical Devices (AEMPS), only aggregated and anonymized data were used, and no information identifying individual participants was accessible to the authors.

### Main outcome measures

Exploratory drug–event statistical signals involving DPP4 inhibitors and SGLT2 inhibitors identified in the community pharmacy dataset and considered candidates for further evaluation in pharmacovigilance or analytical studies.

The shared dataset includes only aggregated drug–event pair counts and derived variables required for signal detection, without any individual-level patient information.

The study considered all DPP4 and SGLT2 inhibitors available, but only signals reaching the predefined detection thresholds in the real-life ambulatory analysis were presented and discussed in the manuscript and Supplement material.

## Results

From the real-life ambulatory study, complete *ad hoc* questionnaires collected were 361, including 974 suspicions of adverse reactions. After process and extract all signals from the real-life ambulatory study, we strictly obtained as positive signal (FDR < 0.05) diarrhea related to metformin (FDR = 0.05), stated in the fact sheets as very frequent and confirming it as a control of the method.

All other signals obtained were FDR > 0.05, so we consider into analysis as early positive signals those with FDR≤0.10, high specificity (Sp≥0.99) and FNR < 49%. (Table 1).

Several of these exploratory signals were based on a small number of observations and should therefore be interpreted with caution as preliminary findings requiring confirmation in larger datasets or analytic study designs.

For descriptive purposes, the exploratory signals were grouped according to whether they were already described in product information and whether similar events were reported for related active ingredients.

### Early safety signals stated in fact sheets

**Group 1.** Early signals to follow up in agreement with ADRs, stated as frequent in fact sheets and also stated for other active ingredients of their group: urinary tract infections related to dapagliflozin.

**Group 2.** Early signals to follow up in agreement with ADR stated as frequent in fact sheets; but of unknown frequency with other active ingredients of their group: exanthematic eruption related with alogliptin, not reported for other gliptins.

**Group 3.** Early signals to follow up in agreement with ADR stated as uncommon in fact sheets and not reported with other active ingredients of the group: dry mouth related to sitagliptin (reported with the appreciation of occurrence if combined with insulin) not reported for other gliptins.

**Group 4.** Early signals to follow up, stated as infrequent in fact sheets, and not reported for other active ingredients of the group: urticaria related with canagliflozin.

### Early safety signals not stated in fact sheets

**Group 5.** Early signals to follow up and not reported as ADR in fact sheets but stated for other active ingredients of the group (these may be masked by common symptoms of T2D): pruritus related with canagliflozin

**Group 6.** Early signals to follow up and not reported as ADR in fact sheets (they may be masked by common symptoms of T2D): asthenia related to empagliflozin and hyperglycemia related with dapagliflozin.

**Group 7.** Early signals to follow up and not reported as ADR in fact sheets: renal impairment and bone fracture related with linagliptin.

According to the threshold, three signals obtained that also are stated in fact sheets confirm the methodology: sitagliptin-dry mouth (FDR = 0.059), dapagliflozin-urinary tract infections (FDR = 0.096), and alogliptin-exanthematic eruptions (FDR = 0.100).

We observed highlighted signals to follow up and not reported in the Summaries of Product Characteristics for the pairs empagliflozin-asthenia (FDR = 0.067), linagliptin-bone fracture (FDR = 0.077), linagliptin-renal impairment (FDR = 0.082), and dapagliflozin-hyperglycemia (FDR = 0.088), and canagliflozin-pruritus (FDR = 0.092).

From the national accumulative reporting database, we obtained 6,602 suspicions of adverse events in 2022 and 7,449 in 2024. If we contrast these ambulatory results of the safety traces to follow up with those obtained from the analysis of the national reporting database (Fig 2), we can observe that some of them did not appear in the analysis of 2022 -despite that some of them are already stated in the fact sheet-, but they become early positive signals in the analysis of 2024. So, it led to hypothesizing that ambulatory early signals studies might have high ability to detect early positive signals or safety traces to follow up complementing spontaneous reporting.

Coincident new signals in spontaneous reporting and signals obtained from Real World Data detected two years earlier include dry mouth related to sitagliptin (stated as uncommon in fact sheet), pruritus and canagliflozin (not reported as ADR in fact sheets but stated for other active ingredients of the group), and asthenia and empagliflozin (neither reported as ADR in fact sheets, nor for its group).

Signals tending to be positive signals due to their progression and coincidence with early signals in RWD are multiple bone fracture and renal impairment related to linagliptin, and urticaria and canagliflozin.

## Discussion

In this study, we explored whether community pharmacy real-world data could identify early drug–event statistical signals involving DPP4 inhibitors and SGLT2 inhibitors and whether some of these signals would later show convergence with patterns observed in spontaneous reporting data. Our findings support the potential value of pharmacy-based surveillance as a complementary source for early signal generation, while also underscoring the need for cautious interpretation.

It is already known that data about patient-reported adverse events or suspected ADRs obtained through spontaneous notification systems are useful for identifying the risks of severe adverse reactions, but they are not very sensitive to mild and prevalent adverse reactions [27]. Therefore, implementing *ad hoc* questionnaires is therefore useful to obtain information on type A adverse reactions, which are more prevalent and less severe. Interestingly, our results, comparing data from both studies -the follow-up and the spontaneous reporting study- suggest we may have identified early signals worth further investigation.

*Group 1.* This is the case of urinary tract infections (UTI) and dapagliflozin. UTI is an early safety signal to follow up in agreement with ADRs stated as frequent in fact sheets and, also, stated for other gliflozins. Diabetes is considered one of the risk factors for UTI in patients receiving SGLT2i. Further research is needed to understand better the mechanisms underlying this association. Nevertheless, the benefits of SGLT2i generally outweigh the risk of genitourinary infections even among those with risk factors [28]

*Group 2.* We identified exanthematic eruption as other early safety signal to follow up stated as frequent in fact sheets for alogliptin, but of unknown frequency for other DPP4i. In post-marketing reports, sitagliptin, saxagliptin, linagliptin, and alogliptin have been associated with hypersensitivity reactions, including anaphylaxis, angioedema, and more severe blistering skin conditions, including Stevens-Johnson syndrome [2]. However, this ADR may seem to be specific to alogliptin. Recently, some authors reminded that serious hypersensitivity to a specific DPP-4 inhibitor or any component of the formulation is a contraindication for treatment with a DPP-4 inhibitor include. [29]

Alogliptin has been uniquely associated with exanthematous eruptions compared to other DPP4i. This may be related to drug-specific hypersensitivity mechanisms, possibly linked to HLA polymorphisms or excipient differences. Previous case reports and post-marketing data confirm its association with serious skin reactions such as Stevens-Johnson syndrome. [30]

*Group 3.* Dry mouth, an ADR stated as uncommon in fact sheets and not reported with other gliptins, is an early safety signal to follow up for sitagliptin specially reported with if combined with insulin. It is known that T2D patients show a higher prevalence of xerostomia than non-DM patients [31–33]. In our study, dry mouth related to sitagliptin did not appear as a positive signal in spontaneous reporting in 2022 but, it was converted into a positive signal in 2024.

*Group 4.* Finally, among ADRs stated in fact sheets, urticaria is only present in the case of canagliflozin, as infrequent ADR, but not for other gliflozins. Cutaneous reactions are rare but can sometimes be severe enough to require discontinuation of this beneficial class. Our findings are consistent with previous research in Japan, where the use of SGLT2i has been associated with important cutaneous and subcutaneous tissue disorders including severe generalized rash, urticaria, erythema and eczema [34]. These skin reactions were usually observed within the first two weeks of treatment, and even on the first day in some patients. Ipragliflozin was the first SGLT2i to be linked to these reports but other SGLT2i such us dapagliflozin which were later marketed later in Japan, were also involved to a lesser extent due to a lower incidence of occurrence [35]. The mechanism underlying this effect is unclear. It has been hypothesized with the diuretic effect of a SGLT2i which could modify skin hydration in T2D patients [36].

*Group 5.* This set of signals may be masked by common symptoms of T2D. This is the case for pruritus, not reported as ADR in fact sheets of canagliflozin but stated for other gliflozins. Previous reports of pruritus induced by SGLT2i have been rare [37]. SGLT2i most well-known side effects are genital mycotic infections and urinary tract infections (UTIs). Although pruritus is less well known, we highlight in our follow up study this suspected side effect as notable, albeit uncommon, in order to sensitize clinicians to its possibility. [38]

*Group 6*. Group 6 included exploratory signals involving empagliflozin–asthenia and dapagliflozin–hyperglycemia, both of which may be difficult to interpret because similar symptoms can also occur in patients with diabetes irrespective of treatment.

We observed notable signals not reported in the fact sheets for the empagliflozin-asthenia pair. Asthenia is a symptom frequently reported in diabetes patients. In a study conducted in elderly patients, fatigue was 3 times more common in this of type of patients and was found on those with a HBA1C value of 10% [39].

The empagliflozin–asthenia signal became more prominent in the 2024 spontaneous reporting analysis, but this pattern should still be interpreted as signal convergence rather than confirmation of causality.

The dapagliflozin–hyperglycemia association should be interpreted cautiously. Rather than representing a confirmed adverse drug reaction, this statistical signal may reflect treatment failure, disease progression, inadequate glycaemic control, non-adherence, or reduced effectiveness in patients with impaired renal function. Because renal function and other clinical covariates were not available in the analytical dataset, we cannot determine the mechanism underlying this finding. We therefore consider this result to be a hypothesis-generating signal that warrants further evaluation rather than evidence of a direct causal adverse drug reaction.

*Group 7*. Strong candidates for follow up, as they are not reported as ADR in fact sheets, are renal impairment and bone fracture related to linagliptin, but it should be considered that diabetes mellitus can cause or exacerbate these health problems.

Renal impairment is frequent in T2D due to microvascular complications and diabetes treatment, and treatment options for these patients are limited. In comparative studies, linagliptin is a DPP-4 inhibitor with a hepatobiliary route of elimination. In comparative studies, it was noninferior to metformin and sulfonylureas in lowering glycated haemoglobin (HbA1c) and improving glycaemic parameters. It has a hepatobiliary route of elimination and it can be used throughout all stages of renal impairment without dose adjustments [40]. DPP4i as drug class seems to have a renal protective effect [41] and linagliptin could have a neutral effect in T2D patients with moderate or severe chronic kidney disease [42]. Consequently, we cannot rule out that patients with renal failure are being prescribed linagliptin preferentially over other therapies. A higher prescription of linagliptin in renal failure patients could explain the emergence of this signal, and further validate the quality of the data obtained from the follow-up study. In our results, the renal failure-canagliflozin pair did not appear as a signal in the follow-up study or in the 2022 spontaneous reporting analysis but it subsequently becomes a positive signal in 2024.

In our analysis of the RWD study, we found a possible risk of bone fracture related to the use of linagliptin. In addition, in the analysis of spontaneous notification data tends to become a positive signal. The major clinical trials did not show a significant increase in the risk of fracture for linagliptin compared to other gliptins. However, alogliptin may be associated with a lower risk of bone fracture compared to placebo, linagliptin, or saxagliptin [43]. Currently, therapies that are based on incretin effect, such as DPP-4i and Glucagon-like peptide-1 receptor agonists (GLP-1RAs) might have positive effects on bone health by promoting bone formation [44]. It is known that incretins like GLP-1 and GIP have receptors on bone cells, suggesting a potential impact on bone metabolism [45], but the risk or protective role of DPP4i in the incidence of fractures in T2D patients has not been conclusively established. In a recent meta-analysis, authors suggest a significant association of the low risk of fracture with the use of SGLT2i (in combination or alone), followed by GLP-1RA, and DPP4i [46]. Due to gliptins appear to be the group the least protective effect, it could be valuable to conduct an exhaustive analysis by active ingredient to detect risk disproportion within this pharmacological group.

However, we must consider the heightened probability of fracture can represent a significant complication for T2D patients when evaluating this signal. Some studies have shown that patients with T2D have a higher risk of fractures even when bone mineral density (BMD) is not significantly reduced, indicating a potential discrepancy between BMD and bone strength in this population [47]. Many studies have suggested that a deteriorated bone microenvironment, rather than a decreased BMD, may be the key factor influencing bone fragility in T2D patients. In any case, we cannot rule out that linagliptin presents a higher risk of bone fracture than other hypoglycemic drugs and we should continue to monitor this potential signal in the future.

### Limitations of the study

This study has several limitations. First, the pharmacy component was based on a multicentre cross-sectional survey with retrospective 6-month recall, which does not establish temporality or causality and may be affected by recall bias. Second, participation was voluntary, which may have introduced selection bias. Third, the restriction to single‑agent antidiabetic therapy and exclusion of non-antidiabetic concomitant treatments into analysis improve interpretability of drug–event signals but limits generalizability to routine clinical practice, where combination therapy is common. Fourth, the disproportionality analysis was performed on drug–event pair data and did not allow adjustment for individual-level covariates such as age, sex, diabetes duration, renal function, comorbidities, dose, treatment duration, or concomitant medications; therefore, some associations may reflect confounding by indication or prescribing patterns rather than drug-specific effects. Fifth, several exploratory signals were based on small numbers of observations, which may reduce estimate stability. Sixth, the FEDRA database provides aggregated spontaneous reporting information and does not permit patient-level comparisons across the two data sources. Finally, signal detection studies identify disproportionality patterns and are inherently hypothesis-generating; they do not confirm adverse drug reactions or biological mechanisms.

### Strengths of the study

In our study, we compared data from a pharmacy network study conducted in 2021 with spontaneous adverse drug reaction reports collected in 2022 and 2024. This comparison allowed us to confirm and validate the safety signals initially observed in 2021.

While we did not perform mechanistic investigations, this approach of using data from different sources and time points helps to strengthen the evidence for the signals by observing their persistence over time.

It is important to note that our study lies in the rapid collection of real-world data (RWD) through pharmacy networks, which allows us to anticipate signals before they appear in spontaneous adverse event reports.

We acknowledge that signal mining primarily identifies statistical correlations and does not establish biological causality. In real-world clinical practice, global introspection methods such as the World Health Organization-Uppsala Monitoring Centre (WHO-UMC) scale [48] or algorithms -that are simple and mostly questionnaire-based methods in which scores are assigned to answers to its questions- such as the the most commonly available criteria in Naranjo algorithm [49] used worldwide.

While our study focused on signal detection from spontaneous reports, we agree that integrating such clinical causality assessments for future research would enhance the understanding of drug-event relationships.

## Conclusions

Community pharmacy-based real-world data collection can support the early detection of potential safety signals, particularly for mild or moderate events that may be underrepresented in traditional spontaneous reporting systems. Our findings suggest that this approach may complement, and in some cases anticipate, spontaneous reporting. Signals identified close to the exploratory threshold and not described in product information should therefore be regarded as candidates for monitoring and further evaluation, rather than as confirmed adverse drug reactions.

In our dataset, signals involving dry mouth with sitagliptin and asthenia with empagliflozin were identified in the community pharmacy data before analogous patterns became more prominent in the spontaneous reporting database. Other findings, including renal impairment, bone fracture, skin reactions, or hyperglycemia-related signals, require cautious interpretation because they may also reflect the underlying disease, clinical context, or prescribing patterns.

## Supporting information

S1 File Data collection questionnaire. (PDF)

S1 Dataset Minimal anonymized real world data (RWD). Aggregated drug–event pairs and results of BCPNN analyses.
(XLSX)

S2 File Details of the statistical package and BCPNN implementation. (PDF)

S1 Table Comparative table of follow-up drug-event changes (2022 vs 2024) for early potential safety signals obtained in spontaneous reports. Event effect (PT), Preferred Term MedDRA; count (n), number of pairs active-ingredient-even effect; FDR, false discovery rate; FDR 2024−2022, difference in FDR values in 2024 compared to 2022; Interpretation, combination of positive, null or negative result in FDR difference and changes since 2022.
(PDF)

## References

[1] Stein SA, Lamos EM, Davis SN. A review of the efficacy and safety of oral antidiabetic drugs. Expert Opin Drug Saf. 2013;12(2):153–75. doi: 10.1517/14740338.2013.752813 23241069 PMC3977601

[2] Gilbert MP, Pratley RE. GLP-1 Analogs and DPP-4 Inhibitors in Type 2 Diabetes Therapy: Review of Head-to-Head Clinical Trials. Front Endocrinol (Lausanne). 2020;11:178. doi: 10.3389/fendo.2020.00178 32308645 PMC7145895

[3] Saisho Y. SGLT2 Inhibitors: The Star in the Treatment of Type 2 Diabetes?. Diseases. 2020;8(2):14. doi: 10.3390/diseases8020014 32403420 PMC7349723

[4] Xu B, Li S, Kang B, Zhou J. The current role of sodium-glucose cotransporter 2 inhibitors in type 2 diabetes mellitus management. Cardiovasc Diabetol. 2022;21(1):83. doi: 10.1186/s12933-022-01512-w 35614469 PMC9134641

[5] Qiu M, Ding L-L, Zhang M, Zhou H-R. Safety of four SGLT2 inhibitors in three chronic diseases: A meta-analysis of large randomized trials of SGLT2 inhibitors. Diab Vasc Dis Res. 2021;18(2):14791641211011016. doi: 10.1177/14791641211011016 33887983 PMC8481734

[6] Fitchett D. A safety update on sodium glucose co-transporter 2 inhibitors. Diabetes Obes Metab. 2019;21 Suppl 2:34–42. doi: 10.1111/dom.13611 31081590

[7] Hauben M, Madigan D, Gerrits CM, Walsh L, Van Puijenbroek EP. The role of data mining in pharmacovigilance. Expert Opin Drug Saf. 2005;4(5):929–48. doi: 10.1517/14740338.4.5.929 16111454

[8] Ahmed I, Haramburu F, Fourrier-Réglat A, Thiessard F, Kreft-Jais C, Miremont-Salamé G, et al. Bayesian pharmacovigilance signal detection methods revisited in a multiple comparison setting. Stat Med. 2009;28(13):1774–92. doi: 10.1002/sim.3586 19360795

[9] Edwards IR, Aronson JK. Adverse drug reactions: definitions, diagnosis, and management. Lancet. 2000;356(9237):1255–9. doi: 10.1016/S0140-6736(00)02799-9 11072960

[10] WHO-UMC. What is a signal. https://who-umc.org/signal-work/what-is-a-signal/. Accessed 2024 December 13.

[11] Patadia VK, Coloma P, Schuemie MJ, Herings R, Gini R, Mazzaglia G, et al. Using real-world healthcare data for pharmacovigilance signal detection - the experience of the EU-ADR project. Expert Rev Clin Pharmacol. 2015;8(1):95–102. doi: 10.1586/17512433.2015.992878 25487079

[12] Bate A, Evans SJW. Quantitative signal detection using spontaneous ADR reporting. Pharmacoepidemiol Drug Saf. 2009;18(6):427–36. doi: 10.1002/pds.1742 19358225

[13] Lindquist M, Ståhl M, Bate A, Edwards IR, Meyboom RH. A retrospective evaluation of a data mining approach to aid finding new adverse drug reaction signals in the WHO international database. Drug Saf. 2000;23(6):533–42. doi: 10.2165/00002018-200023060-00004 11144660

[14] Spanish Agency of Medicines and Medical Devices (AEMPS). Pharmacovigilance of Medicines for Human Use. Report on Suspected Reported Adverse Reactions to Medicinal Products for Human Use or Adverse Events Occurring After Vaccination. https://www.aemps.gob.es/medicamentos-de-uso-humano/farmacovigilancia-de-medicamentos-de-uso-humano/

[15] International Council for Harmonisation of Technical Requirements for Pharmaceuticals for Human Use ICH. MedDRA Hierarchy. https://www.ich.org/page/meddra. Accessed 2024 December 13.

[16] Bate A, Lindquist M, Edwards IR, Olsson S, Orre R, Lansner A, et al. A Bayesian neural network method for adverse drug reaction signal generation. Eur J Clin Pharmacol. 1998;54(4):315–21. doi: 10.1007/s002280050466 9696956

[17] Roux E, Thiessard F, Fourrier A, Bégaud B, Tubert-Bitter P. Evaluation of statistical association measures for the automatic signal generation in pharmacovigilance. IEEE Trans Inf Technol Biomed. 2005;9(4):518–27. doi: 10.1109/titb.2005.855566a 16379369

[18] Bate A, Lindquist M, Orre R, Edwards IR, Meyboom RHB. Data-mining analyses of pharmacovigilance signals in relation to relevant comparison drugs. Eur J Clin Pharmacol. 2002;58(7):483–90. doi: 10.1007/s00228-002-0484-z 12389072

[19] Bate A, Lindquist M, Edwards IR. The application of knowledge discovery in databases to post-marketing drug safety: example of the WHO database. Fundam Clin Pharmacol. 2008;22(2):127–40. doi: 10.1111/j.1472-8206.2007.00552.x 18248442

[20] Bate A. Bayesian confidence propagation neural network. Drug Saf. 2007;30(7):623–5. doi: 10.2165/00002018-200730070-00011 17604417

[21] Zorych I, Madigan D, Ryan P, Bate A. Disproportionality methods for pharmacovigilance in longitudinal observational databases. Stat Methods Med Res. 2013;22(1):39–56. doi: 10.1177/0962280211403602 21878461

[22] Norén GN, Edwards IR. Modern methods of pharmacovigilance: detecting adverse effects of drugs. Clin Med (Lond). 2009;9(5):486–9. doi: 10.7861/clinmedicine.9-5-486 19886114 PMC4953463

[23] WHO Collaborating Centre for Drug Statistics Methodology. ATC/DDD Index. https://atcddd.fhi.no/atc_ddd_methodology/who_collaborating_centre. Accessed 2024 December 13.

[24] Jimenez-Serrania MI. New strategy for early detection of drug-associated safety signals: the case of rosiglitazone. Spain.

[25] Treceño-Lobato C, Jiménez-Serranía M-I, Martínez-García R, Corzo-Delibes F, Martín Arias LH. New Anticoagulant Agents: Incidence of Adverse Drug Reactions and New Signals Thereof. Semin Thromb Hemost. 2019;45(2):196–204. doi: 10.1055/s-0038-1657783 29864777

[26] Jimenez-Serrania M-I, Treceño-Lobato C. Influence of Concomitant Treatments under Anticoagulants and Statins in Detecting Signals of Adverse Drug Reactions. Semin Thromb Hemost. 2019;45(8):837–45. doi: 10.1055/s-0039-1695734 31563128

[27] Carvajal A, Sáinz M, Velasco V, García Ortega P, Treceño C, Martín Arias LH, et al. Emergency contraceptive pill safety profile. Comparison of the results of a follow-up study to those coming from spontaneous reporting. Pharmacoepidemiol Drug Saf. 2015;24(1):93–7. doi: 10.1002/pds.3725 25408302

[28] Kittipibul V, Cox ZL, Chesdachai S, Fiuzat M, Lindenfeld J, Mentz RJ. Genitourinary Tract Infections in Patients Taking SGLT2 Inhibitors. J Am Coll Cardiol. 2024;83(16):1568–78. doi: 10.1016/j.jacc.2024.01.040 38631776

[29] Zaresharifi S, Niroomand M, Borran S, Dadkhahfar S. Dermatological side effects of dipeptidyl Peptidase-4 inhibitors in diabetes management: a comprehensive review. Clin Diabetes Endocrinol. 2024;10(1):6. doi: 10.1186/s40842-024-00165-w 38523307 PMC10962164

[30] Chen X-W, He Z-X, Zhou Z-W, Yang T, Zhang X, Yang Y-X, et al. An update on the clinical pharmacology of the dipeptidyl peptidase 4 inhibitor alogliptin used for the treatment of type 2 diabetes mellitus. Clin Exp Pharmacol Physiol. 2015;42(12):1225–38. doi: 10.1111/1440-1681.12469 26218204

[31] Mauri-Obradors E, Estrugo-Devesa A, Jané-Salas E, Viñas M, López-López J. Oral manifestations of Diabetes Mellitus. A systematic review. Med Oral Patol Oral Cir Bucal. 2017;22(5):e586–94. doi: 10.4317/medoral.21655 28809366 PMC5694181

[32] Javed F, Sundin U, Altamash M, Klinge B, Engström P-E. Self-perceived oral health and salivary proteins in children with type 1 diabetes. J Oral Rehabil. 2009;36(1):39–44. doi: 10.1111/j.1365-2842.2008.01895.x 18976260

[33] Sandberg GE, Sundberg HE, Wikblad KF. A controlled study of oral self-care and self-perceived oral health in type 2 diabetic patients. Acta Odontol Scand. 2001;59(1):28–33. doi: 10.1080/000163501300035742 11318042

[34] Yabe D, Nishikino R, Kaneko M, Iwasaki M, Seino Y. Short-term impacts of sodium/glucose co-transporter 2 inhibitors in Japanese clinical practice: considerations for their appropriate use to avoid serious adverse events. Expert Opin Drug Saf. 2015;14(6):795–800. doi: 10.1517/14740338.2015.1034105 25851664

[35] Sakaeda T, Kobuchi S, Yoshioka R, Haruna M, Takahata N, Ito Y, et al. Susceptibility to serious skin and subcutaneous tissue disorders and skin tissue distribution of sodium-dependent glucose co-transporter type 2 (SGLT2) inhibitors. Int J Med Sci. 2018;15(9):937–43. doi: 10.7150/ijms.22224 30008607 PMC6036094

[36] Tezuka Y, Sekine O, Hirano A, Hanada Y, Nakanishi I, Ariga M, et al. A Prospective, Open-Label Short-Term Pilot Study on Modification of the Skin Hydration Status During Treatment With a Sodium-Glucose Cotransporter-2 Inhibitor. Diabetes Ther. 2021;12(1):431–40. doi: 10.1007/s13300-020-00950-7 33108650 PMC7843859

[37] Vasapollo P, Cione E, Luciani F, Gallelli L. Generalized Intense Pruritus During Canagliflozin Treatment: Is it an Adverse Drug Reaction?. Curr Drug Saf. 2018;13(1):38–40. doi: 10.2174/1574886311666160405110515 27048192

[38] Ali K, Mohammed SR, Deonarine R, Teelucksingh S. Sodium-glucose co-transporter-2 inhibitor-induced pruritus: itching for answers. Cureus. 2021;13(8):e17573. doi: 10.7759/cureus.17573 PMC8480444 34646628

[39] Esen I, Akturk Esen S, Demirci H. Fatigue and depression in elderly patients with poorly controlled diabetes. Medicine (Baltimore). 2022;101(45):e31713. doi: 10.1097/MD.0000000000031713 36397324 PMC9666150

[40] Gallwitz B. Management of patients with type 2 diabetes and mild/moderate renal impairment: profile of linagliptin. Ther Clin Risk Manag. 2015;11:799–805. doi: 10.2147/TCRM.S67076 25999728 PMC4437596

[41] Bae JH, Kim S, Park EG, Kim SG, Hahn S, Kim NH. Effects of Dipeptidyl Peptidase-4 Inhibitors on Renal Outcomes in Patients with Type 2 Diabetes: A Systematic Review and Meta-Analysis. Endocrinol Metab (Seoul). 2019;34(1):80–92. doi: 10.3803/EnM.2019.34.1.80 30912341 PMC6435854

[42] Hanssen NM, Jandeleit-Dahm KA. Dipeptidyl peptidase-4 inhibitors and cardiovascular and renal disease in type 2 diabetes: What have we learned from the CARMELINA trial?. Diab Vasc Dis Res. 2019;16(4):303–9. doi: 10.1177/1479164119842339 31018682 PMC6613297

[43] Yang J, Huang C, Wu S, Xu Y, Cai T, Chai S, et al. The effects of dipeptidyl peptidase-4 inhibitors on bone fracture among patients with type 2 diabetes mellitus: A network meta-analysis of randomized controlled trials. PLoS One. 2017;12(12):e0187537. doi: 10.1371/journal.pone.0187537 29206832 PMC5716604

[44] Psachna S, Chondrogianni ME, Stathopoulos K, Polymeris A, Chatzigeorgiou A, Chronopoulos E, et al. The effect of antidiabetic drugs on bone metabolism: a concise review. Endocrine. 2025;87(3):907–19. doi: 10.1007/s12020-024-04070-1 39402366

[45] Sanz C, Vázquez P, Blázquez C, Barrio PA, Alvarez MDM, Blázquez E. Signaling and biological effects of glucagon-like peptide 1 on the differentiation of mesenchymal stem cells from human bone marrow. Am J Physiol Endocrinol Metab. 2010;298(3):E634-43. doi: 10.1152/ajpendo.00460.2009 20040695

[46] Mostafa MEA, Alrasheed T. Risk of bone fracture by using dipeptidyl peptidase-4 inhibitors, glucagon-like peptide-1 receptor agonists, or sodium-glucose cotransporter-2 inhibitors in patients with type 2 diabetes mellitus: a network meta-analysis of population-based cohort studies. Front Endocrinol (Lausanne). 2024;15:1410883. doi: 10.3389/fendo.2024.1410883 39464183 PMC11502341

[47] Huang L, Zhong W, Liang X, Wang H, Fu S-E, Luo Z. Meta-Analysis on the Association Between DPP-4 Inhibitors and Bone Mineral Density and Osteoporosis. J Clin Densitom. 2024;27(1):101455. doi: 10.1016/j.jocd.2023.101455 38101289

[48] World Health Organization (WHO)-Uppsala Monitoring Centre. The use of the WHO-UMC system for standardized case causality assessment. http://www.who-umc.org/Graphics/24734.pdf. Accessed 2025 July 14.

[49] Naranjo CA, Busto U, Sellers EM, Sandor P, Ruiz I, Roberts EA, et al. A method for estimating the probability of adverse drug reactions. Clin Pharmacol Ther. 1981;30(2):239–45. doi: 10.1038/clpt.1981.154 7249508

