# Supplementary material for: Early potential safety signals for gliptins and gliflozins using real-world pharmacy data compared to spontaneous reporting
Source: PLoS One. 2026 Jun 25;21(6):e0352399. doi: 10.1371/journal.pone.0352399 (PMC13298747; doi:10.1371/journal.pone.0352399)
Supplement: S1 File — (PDF) [file pone.0352399.s001.pdf]

# S1 File. Data collection questionnaire

**1) Descriptive Data:**

a. Age (numeric)

b. Sex (coded as M: male, F: female)

c. Medications (national drug code); ATC code; active ingredient

d. Dosing schedule (X times/day)

e. Dosing interval (every X hours)

f. Diagnosed conditions (coded using ICD-10-ES Diagnoses 2018, eCIE-Maps - ICD-10-ES Diagnoses: https://eciemaps.msssi.gob.es/ecieMaps/browser/metabuscador.html)

g. As-needed (PRN) medications (national drug code)

h. Most recent HbA1c value (patient reported – if available)

---

**2) Adverse Reactions Reported in the Past Six Months:**

**Hematologic:**

1. Hematoma
2. Epistaxis
3. Ecchymosis
4. Lymphocytopenia
5. Increased hematocrit
6. Anemia
    - 6.1 Iron-deficiency
    - 6.2 Megaloblastic

**Nervous System:**

1. Vertigo
2. Headache
3. Dizziness
4. Syncope

**Dermatologic:**

1. Angioedema
2. Dermatitis
3. Pruritus, urticaria
4. Exanthematous rash
5. Bullous pemphigoid

**Cardiovascular:**

1. Heart failure
2. Hypotension
3. Orthostatic hypotension
4. Hypovolemia
5. Edema

**Musculoskeletal:**

1. Bone fracture
2. Osteoporosis
3. Musculoskeletal pain, limb pain

**Gastrointestinal:**

1. Nausea and vomiting
2. Abdominal pain
3. Diarrhea
4. Dyspepsia
5. Polydipsia
6. Dry mouth
7. Acute pancreatitis

**Metabolic:**

1. Hypoglycemia
2. Hypercholesterolemia
3. Hyperkalemia
4. Hyperphosphatemia
5. Hypermagnesemia
6. Diabetic ketoacidosis
7. Dehydration

**Infectious:**

1. Respiratory infection
2. Urinary tract infection
3. Gastroenteritis

**Genitourinary:**

1. Polyuria, pollakiuria, nocturia, or urgency
2. Increased serum creatinine
3. Increased blood urea nitrogen
4. Renal insufficiency

**Allergic:**

1. Erythema
2. Pruritus, urticaria
3. Angioedema

**General:**

1. Asthenia
2. Malleolar edema
3. Other health problems experienced in the last six months
