# Supplementary material for: Early potential safety signals for gliptins and gliflozins using real-world pharmacy data compared to spontaneous reporting
Source: PLoS One. 2026 Jun 25;21(6):e0352399. doi: 10.1371/journal.pone.0352399 (PMC13298747; doi:10.1371/journal.pone.0352399)
Supplement: S2 File — (PDF) [file pone.0352399.s003.pdf]

S2 File. Details of the statistical package and BCPNN implementation

IC Formula

The core statistic used in BCPNN method is the Information Component (IC), which quantifies the strength of association between a drug (i) and an adverse event (j).

The IC is calculated as:

$$IC_{ij} = \log_2(p_{ij} / (p_i * p_j))$$

where:

- p_{ij}: joint probability of observing the drug–event combination,
- p_i: marginal probability of the drug,
- p_j: marginal probability of the event.

This formulation compares the observed reporting rate of a drug–event pair to what would be expected under independence.

A confidence interval is then calculated as:

$$IC_{lower} = IC - z_\alpha * \mathrm{sqrt}(VICb)$$

where,

IC (Information Component): Measures the disproportionality between the observed and expected counts of a drug–event combination.

VICb: The posterior variance of the IC, which reflects the uncertainty of the IC estimate. It incorporates both the observed data and the Bayesian prior.

Z_α: The z-score corresponding to the desired confidence level (e.g., 1.96 for a 95% credibility interval).

PhViD R Package (Ismaïl Ahmed & Antoine Poncet)

The PhViD-package proposes Bayesian confidence propagation neural network (Bate et al. 1998, Noren et al. 2006) extended to the multiple comparison setting, the main pharmacovigilance signal detection methods. These functions can be used as standard R functions or through an user friendly interface (PhViD.gui). For the frequentist methods, the package requires the LBE procedure that can be downloaded from the Bioconductor website http://bioconductor.org/packages/2.3/bioc/html/LBE.html.

Usage

BCPNN(DATABASE, RR0 = 1, MIN.n11 = 1, DECISION = 1, DECISION.THRES = 0.05, RANKSTAT = 1, MC = **FALSE**, NB.MC = 10000)

Arguments

**DATABASE**	Object returned by the function as.PhViD.
**RR0**	Value of the tested risk. By default, RR0=1.
**MIN.n11**	Minimum number of notifications for a couple to be potentially considered as a signal. By default, MIN.n11 = 1.
**DECISION**	Decision rule for the signal generation based on<br>1 = FDR (Default value)<br>2 = Number of signals<br>3 = Ranking statistic. See RANKSTAT
**DECISION.THRES**	Threshold for DECISION. Ex 0.05 for FDR (DECISION=1).
**RANKSTAT**	Statistic used for ranking the couples:<br>1 = Posterior probability of the null hypothesis<br>2 = 2.5% quantile of the posterior distribution of IC.
**MC**	If MC=TRUE, the statistic of interest (see RANKSTAT) is calculated by Monte Carlo simulations which can be very long. If MC=FALSE, IC is

approximated by a normal distribution (which can be very crude for small counts).

NB.MC If MC=TRUE, NB.MC indicates the number of Monte Carlo simulations to be done

The BCPNN method is based on the calculation of the Information Component IC. If MC = FALSE, the bayesian model used is the beta-binomial proposed by Bate et al. (1998). The statistic of interest (see RANKSTAT) is calculated by the normal approximation made in Bate et al. (1998) with the use of the exact expectation and variance proposed by Gould (2003). If MC = TRUE, the model is based on the Dirichlet-multinomial model proposed more recently in Noren et al. (2006). In this case, the statistic of interest is calculated by Monte Carlo simulations.

Value

ALLSIGNALS Data.frame summarizing the results of all couples with at least MIN.n11 notifications ordered by RANKSTAT. It contains notably the labels, the cell counts, the expected counts (*n1. * n.1 / N*, see as.PhViD), RANKSTAT, the ratios(count/expected count), the marginal counts and the estimations of FDR, FNR, Se et Sp. If RANKSTAT!=1, the last column is the posterior probability of the null hypothesis.

SIGNALS Same Data.frame as ALLSIGNALS but restricted to the list of generated signals.

NB.SIGNALS Number of generated signals.

INPUT.PARAM Parameters entered in the function.

Details of algorithm performed in the study:

- value of the relative risk (RR) proven to be higher than 1 (RR<1);
- minimum number of cases per pair [drug-adverse reaction] to be potentially considered as a signal (N=1);
- rule of decision for the generation of signals: false discovery rate (FDR);
- limit or threshold for the decision rule: FDR>0.05;
- statistics used for ordering the drug-event pairs: posterior probability of the null hypothesis (post.H0); - calculation of the distribution of the statistic of interest: by approximation to the normal distribution.
- and using empirical estimation through Monte Carlo simulations (NB.MC=10000).

The estimator of FDR<0.05 and specificity (Sp) ≥0.99 are considered to interpret the results. Sensitivity (Se) values are typically low in the BCPNN approach, Se ≥0.20 is considered as reference.

The estimator FDR assures that at least 95% of the signals detected are positive (only 5% of false positives). Moreover, if the estimator of false negatives (FNR) is 50% or lower, it implicates that, at least, half of the signals rejected are effectively negative. In the results presented, all the FNR were lower than 49%.

Key References

Ahmed I, Haramburu F, Fourrier-Réglat A, Thiessard F, Kreft-Jais C, Miremont-Salamé G, Bégaud B, Tubert-Bitter P. Bayesian pharmacovigilance signal detection methods revisited in a multiple comparison setting. Stat Med. 2009 Jun 15;28(13):1774-1792.

Bate A, Lindquist M, Edwards IR, Olsson S, Orre R, Lansner A, De Freitas RM, A Bayesian Neural Network Method for Adverse Drug Reaction Signal Generation European Journal of Clinical Pharmacology, 1998, 54, 315-321.

Gould AL, Practical Pharmacovigilance Analysis Strategies Pharmacoepidemiology and Drug Safety, 2003, 12, 559-574

Noren, GN, Bate A, Orre R, Edwards IR, Extending the methods used to screen the WHO drug safety database towards analysis of complex associations and improved accuracy for rare events Statistics in Medicine, 2006, 25, 3740-3757.
