# Supplementary material for: Early potential safety signals for gliptins and gliflozins using real-world pharmacy data compared to spontaneous reporting
Source: PLoS One. 2026 Jun 25;21(6):e0352399. doi: 10.1371/journal.pone.0352399 (PMC13298747; doi:10.1371/journal.pone.0352399)
Supplement: S1 Table — Event effect (PT), Preferred Term MedDRA; count (n), number of pairs active-ingredient-even effect; FDR, false discovery rate; FDR 2024−2022, difference in FDR values in 2024 compared to 2022; Interpretation, combination of positive, null or negative result in FDR difference and changes since 2022. (PDF) [file pone.0352399.s004.pdf]

S1 Table. Comparative table of follow-up drug-event changes (2022 vs 2024) for early potential safety signals obtained in spontaneous reporting.

Dry mouth							
**Active ingredient**	**Event Effect (PT)**	**Count (n)**	**FDR 2022**	**Count (n)**	**FDR 2024**	**FDR 2024-2022**	**Interpretation**
canagliflozin	Dry mouth	2	0.311	2	0.305	-0.006	no signal but trend toward threshold
dapagliflozin	Dry mouth	17	0.000	20	0.000	0.000	remains a signal
empagliflozin	Dry mouth	6	0.290		No signal		follow-up lost
linagliptin	Dry mouth	1	0.360	1	0.360	0.000	remains not a signal
metformin	Dry mouth	4	0.401	7	0.390	-0.011	no signal but trend toward threshold
sitagliptin	Dry mouth	5	0.109	7	**0.021**	-0.088	**HAS BECOME A SIGNAL**
Asthenia							
**Active ingredient**	**Event Effect (PT)**	**Count (n)**	**FDR 2022**	**Count (n)**	**FDR 2024**	**FDR 2024-2022**	**Interpretation**
canagliflozin	Asthenia	1	0.380	1	0.375	-0.005	no signal but trend toward threshold
dapagliflozin	Asthenia	6	0.360	10	0.357	-0.003	no signal but trend toward threshold
empagliflozin	Asthenia	9	0.348	14	**0.002**	-0.346	**HAS BECOME A SIGNAL**
linagliptin	Asthenia	3	0.348	4	0.346	-0.002	no signal but trend toward threshold
metformin	Asthenia	43	0.068	48	0.265	0.197	remains not a signal
sitagliptin	Asthenia	5	0.347	6	0.347	0.000	remains not a signal
vildagliptin	Asthenia	2	0.345	2	0.348	0.003	remains not a signal
Bone fracture							
**Active ingredient**	**Event Effect (PT)**	**Count (n)**	**FDR 2022**	**Count (n)**	**FDR 2024**	**FDR 2024-2022**	**Interpretation**
dapagliflozin	Femur fracture	1	0.209	1	0.230	0.021	remains not a signal

empagliflozin	Hand fracture	1	0.285	1	0.251	-0.034	no signal but trend toward threshold
linagliptin	Spinal fracture	1	0.164	1	0.169	0.005	remains not a signal
linagliptin	Multiple fractures	1	0.164	1	0.160	-0.004	no signal but trend toward threshold
linagliptin	Ankle fracture	1	0.200	1	0.218	0.018	remains not a signal
linagliptin	Hand fracture	1	0.237	1	0.245	0.008	remains not a signal
metformin	Ankle fracture	1	0.331	1	0.330	-0.001	no signal but trend toward threshold
metformin	Hand fracture	1	0.350	1	0.345	-0.005	no signal but trend toward threshold
canagliflozin	Renal impairment	3	0.058	**3**	**0.036**	-0.022	**HAS BECOME A SIGNAL**
canagliflozin	Renal failure	1	0.361	1	0.350	-0.011	no signal but trend toward threshold
dapagliflozin	Renal disorder		0.217	2	0.108	-0.109	no signal but trend toward threshold
dapagliflozin	Renal failure	15	0.318	8	0.180	-0.138	no signal but trend toward threshold
dapagliflozin	Renal impairment	1	0.369	1	0.362	-0.007	no signal but trend toward threshold
empagliflozin	Renal impairment	6	0.066		No signal		follow-up lost
empagliflozin	Renal failure	7	0.290		No signal		follow-up lost
linagliptin	Renal impairment	2	0.258	2	0.239	-0.019	no signal but trend toward threshold
linagliptin	Renal failure	3	0.280	3	0.282	0.002	remains not a signal
metformin	Renal failure	22	0.292	24	0.250	-0.042	no signal but trend toward threshold
metformin	Renal impairment	6	0.385	7	0.343	-0.042	no signal but trend toward threshold
sitagliptin	Renal impairment	3	0.235	3	0.113	-0.122	no signal but trend toward threshold
sitagliptin	Renal failure	3	0.346	3	0.335	-0.011	no signal but trend toward threshold
vildagliptin	Renal failure	1	0.351	1	0.338	-0.013	no signal but trend toward threshold
sitagliptin	Renal disorder		No signal	1	0.256		new event reported

Hyperglycemia

**Active ingredient**	**Event Effect (PT)**	**Count (n)**	**FDR 2022**	**Count (n)**	**FDR 2024**	**FDR 2024-2022**	**Interpretation**
dapagliflozin	Hyperglycemia	4	0.328	4	0.348	0.020	remains not a signal
empagliflozin	Hyperglycemia	3	0.376		No signal		follow-up lost
linagliptin	Hyperglycemia	5	0.029	5	0.034	0.005	remains a signal
metformin	Hyperglycemia	20	0.284	21	0.254	-0.030	no signal but trend toward threshold
saxagliptin	Hyperglycemia	1	0.249	1	0.247	-0.002	no signal but trend toward threshold
sitagliptin	Hyperglycemia	3	0.335	3	0.320	-0.015	no signal but trend toward threshold
vildagliptin	Hyperglycemia	1	0.345	1	0.334	-0.011	no signal but trend toward threshold
canagliflozin	Pruritus	4	0.358	4	0.351	-0.007	no signal but trend toward threshold

canagliflozin	Vulvovaginal pruritus	4	0.052	5	0.009	**-0.043**	remains a signal AND NOW MORE SIGNIFICANT
canagliflozin	Genital pruritus	2	0.280	2	0.126	-0.154	no signal but trend toward threshold
canagliflozin	Anal pruritus	1	0.153	2	**0.049**	**-0.104**	**HAS BECOME A SIGNAL**
dapagliflozin	Vulvovaginal pruritus	18	0.000	27	0.000	0.000	remains a signal
dapagliflozin	Pruritus	15	0.260	19	0.285	0.025	remains not a signal
dapagliflozin	Genital pruritus	9	0.004	14	0.000	**-0.004**	remains a signal AND NOW MORE SIGNIFICANT
dapagliflozin	Injection site pruritus	1	0.253	1	0.263	0.010	remains not a signal
empagliflozin	Genital pruritus	18	0.000		No signal		follow-up lost
empagliflozin	Vulvovaginal pruritus	14	0.001		No signal		follow-up lost
empagliflozin	Oral pruritus	1	0.232		No signal		follow-up lost
linagliptin	Pruritus	15	0.049	15	0.003	**-0.046**	remains a signal AND NOW MORE SIGNIFICANT
metformin	Pruritus	10	0.383	55	0.371	-0.012	no signal but trend toward threshold
sitagliptin	Rash pruritic	2	0.005	22	0.000	**-0.005**	remains a signal AND NOW MORE SIGNIFICANT
sitagliptin	Pruritus	49	0.192	1	0.208	0.016	remains not a signal
sitagliptin	Pruritus	18	0.243	1	0.242	-0.001	no signal but trend toward threshold
vildagliptin	Pruritus	1	0.027	8	0.024	**-0.003**	remains a signal AND NOW MORE SIGNIFICANT

Urticaria

**Active ingredient**	**Event Effect (PT)**	**Count (n)**	**FDR 2022**	**Count (n)**	**FDR 2024**	**FDR 2024-2022**	**Interpretation**
canagliflozin	Urticaria	3	0.322	4	0.304	-0.018	no signal but trend toward threshold
dapagliflozin	Urticaria	9	0.249	11	0.336	0.087	remains not a signal
dapagliflozin	Vesicular urticaria	1	0.217	1	0.263	0.046	remains not a signal
empagliflozin	Urticaria	3	0.397	9	0.104	-0.293	no signal but trend toward threshold
linagliptin	Urticaria	3	0.344	4	0.340	-0.004	no signal but trend toward threshold
metformin	Urticaria	28	0.366	30	0.384	0.018	remains not a signal
sitagliptin	Urticaria	17	0.000	19	0.000	0.000	remains a signal
vildagliptin	Urticaria	3	0.281	3	0.311	0.030	remains not a signal
alogliptin	Urticaria		No signal	2	0.084		new event reported

metformin	Aquagenic urticaria		No signal	1	0.287		new event reported
metformin	Papular urticaria		No signal	1	0.298		new event reported
metformin	Vesicular urticaria		No signal	1	0.331		new event reported

Urinary tract infections and related

**Active ingredient**	**Event Effect (PT)**	**Count (n)**	**FDR 2022**	**Count (n)**	**FDR 2024**	**FDR 2024-2022**	**Interpretation**
canagliflozin	Urinary tract infection	23	0.000	24	0.000	0.000	remains a signal
canagliflozin	Genitourinary tract infection	1	0.198	1	0.222	0.024	remains not a signal
dapagliflozin	Urinary tract infection	50	0.000	77	0.000	0.000	remains a signal
dapagliflozin	Escherichia urinary tract infection	2	0.129	2	0.095	-0.034	no signal but trend toward threshold
dapagliflozin	Genitourinary tract infection	1	0.251	2	0.109	-0.142	no signal but trend toward threshold
empagliflozin	Urinary tract infection	45	0.000		No signal		follow-up lost
empagliflozin	Urinary tract candidiasis	4	0.038		No signal		follow-up lost
empagliflozin	Escherichia urinary tract infection	3	0.025		No signal		follow-up lost
empagliflozin	Enterococcal urinary tract infection	3	0.039		No signal		follow-up lost
saxagliptin	Urinary tract infection	1	0.315	1	0.282	-0.033	no signal but trend toward threshold
dapagliflozin	Klebsiella urinary tract infection		No signal	1	0.231		new event reported
dapagliflozin	Urinary tract candidiasis		No signal	1	0.233		new event reported
saxagliptin	Urinary tract infection		No signal	1	0.282		new event reported
canagliflozin	Urinary tract fungal infection		No signal	1	0.148		new event reported

Exanthematic eruption (rash) and related

Active ingredient	Event Effect (PT)	Count (n)	FDR 2022	Count (n)	FDR 2024	FDR 2024-2022	Interpretation
canagliflozin	Rash	5	0.099	5	0.112	0.013	remains not a signal
canagliflozin	Genital rash	1	0.245	1	0.212	-0.033	no signal but trend toward threshold
dapagliflozin	Rash	4	0.382	11	0.282	-0.100	no signal but trend toward threshold
dapagliflozin	Erythematous rash	1	0.343	2	0.315	-0.028	no signal but trend toward threshold
dapagliflozin	Genital rash	1	0.287	1	0.260	-0.027	no signal but trend toward threshold
dapagliflozin	Rash on penis	1	0.250		No signal		follow-up lost
empagliflozin	Rash	6	0.379		No signal		follow-up lost
empagliflozin	Genital rash	2	0.122		No signal		follow-up lost
empagliflozin	Toxic skin eruption	2	0.255	2	0.115	-0.140	no signal but trend toward threshold
empagliflozin	Drug eruption	1	0.323	1	0.280	-0.043	no signal but trend toward threshold
empagliflozin	Erythematous rash	1	0.355	1	0.320	-0.035	no signal but trend toward threshold
empagliflozin	Morbilliform rash	1	0.285	1	0.252	-0.033	no signal but trend toward threshold
empagliflozin	Pruritic rash	1	0.319	1	0.273	-0.046	no signal but trend toward threshold
empagliflozin	Rash on penis	1	0.262		No signal		follow-up lost
linagliptin	Rash	5	0.180	6	0.130	-0.050	no signal but trend toward threshold
linagliptin	Erythematous rash	1	0.308	1	0.311	0.003	remains not a signal
linagliptin	Pustular rash		0.163	1	0.161	-0.002	no signal but trend toward threshold
linagliptin	Toxic skin eruption	1	0.277		0.281	0.004	remains not a signal
metformin	Rash	30	0.342	31	0.360	0.018	remains not a signal
metformin	Erythematous rash	8	0.174	8	0.259	0.085	remains not a signal
metformin	Maculopapular rash	8	0.063	8	0.105	0.042	remains not a signal
metformin	Toxic skin eruption	1	0.383		0.368	-0.015	no signal but trend toward threshold

metformin	Drug eruption	3	0.326		0.326	0.000	remains not a signal
metformin	Fixed Rash	3	0.239		0.248	0.009	remains not a signal
metformin	Pruritic rash	2	0.347	2	0.341	-0.006	no signal but trend toward threshold
metformin	Exfoliative rash	1	0.298	1	0.328	0.030	remains not a signal
metformin	Morbilliform rash	1	0.349	1	0.346	-0.003	no signal but trend toward threshold
metformin	Papular rash	1	0.298	1	0.327	0.029	remains not a signal
saxagliptin	Rash	1	0.276	1	0.268	-0.008	no signal but trend toward threshold
saxagliptin	Toxic skin eruption	1	0.174		0.176	0.002	remains not a signal
sitagliptin	Rash	10	0.021	12	0.007	**-0.014**	remains a signal AND NOW MORE SIGNIFICANT
sitagliptin	Drug eruption	2	0.104		0.110	0.006	remains not a signal
sitagliptin	Toxic skin eruption	2	0.112		0.133	0.021	remains not a signal
sitagliptin	Morbilliform rash	1	0.256	1	0.257	0.001	remains not a signal
sitagliptin	Pruritic rash	1	0.282	1	0.276	-0.006	no signal but trend toward threshold
sitagliptin	Vesicular rash	1	0.187	1	0.210	0.023	remains not a signal
vildagliptin	Rash	3	0.277	3	0.276	-0.001	no signal but trend toward threshold
vildagliptin	Pruritic rash	1	0.244	1	0.243	-0.001	no signal but trend toward threshold
empagliflozin	Exfoliative rash		No signal	1	0.225	0.225	new event reported
empagliflozin	Macular rash		No signal	1	0.225	0.225	new event reported
linagliptin	Macular rash		No signal	1	0.217	0.217	new event reported
linagliptin	Maculopapular rash		No signal	1	0.285	0.285	new event reported
linagliptin	Papular rash		No signal	1	0.217	0.217	new event reported
